# Supplementary material for: Exosome Release by Glucose Deprivation Is Important for the Viability of TSC-Null Cells
Source: Cells. 2022 Sep 14;11(18):2862. doi: 10.3390/cells11182862 (PMC9497210; doi:10.3390/cells11182862)
Supplement: Supplementary file 1 [file cells-11-02862-s001.zip › cells-1853422-supplementary.pdf]

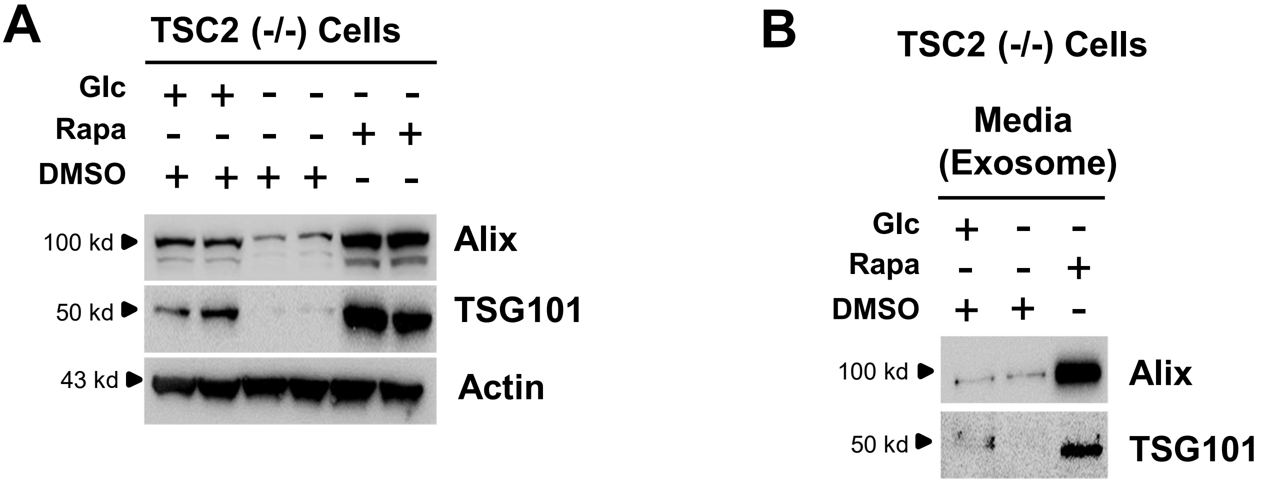

Supple. Figure S1. Effects of exosome release by mTORC1 inhibition in TSC2-null cells.
(A) TSC2 (-/-) MEF cells were cultured with completed media. During serum starvation, cells were cultured in the absence or presence of glucose for 48 h. Rapamycin (100 nM) was treated in the absence of glucose for 24 h. The cells were harvested, lysed, and analyzed by immunoblotting with the indicated antibodies such as Alix, and TSG101. Actin was used as a loading control. (B) TSC2 (-/-) MEF cells were cultured with completed media. During serum starvation, cells were cultured in the absence or presence of glucose for 48 h. Rapamycin (100 nM) was treated in the absence of glucose for 24 h. After the collection of medium from cells, the media were subjected to ultracentrifugation to harvest the exosome fraction, as according to the materials and methods. The samples were analyzed by immunoblotting with antibodies for exosome marker proteins, such as Alix, and TSG101. Glucose (Glc), Rapamycin (Rapa), Dimethyl Sulfoxide (DMSO).

Supplemental Figure S1

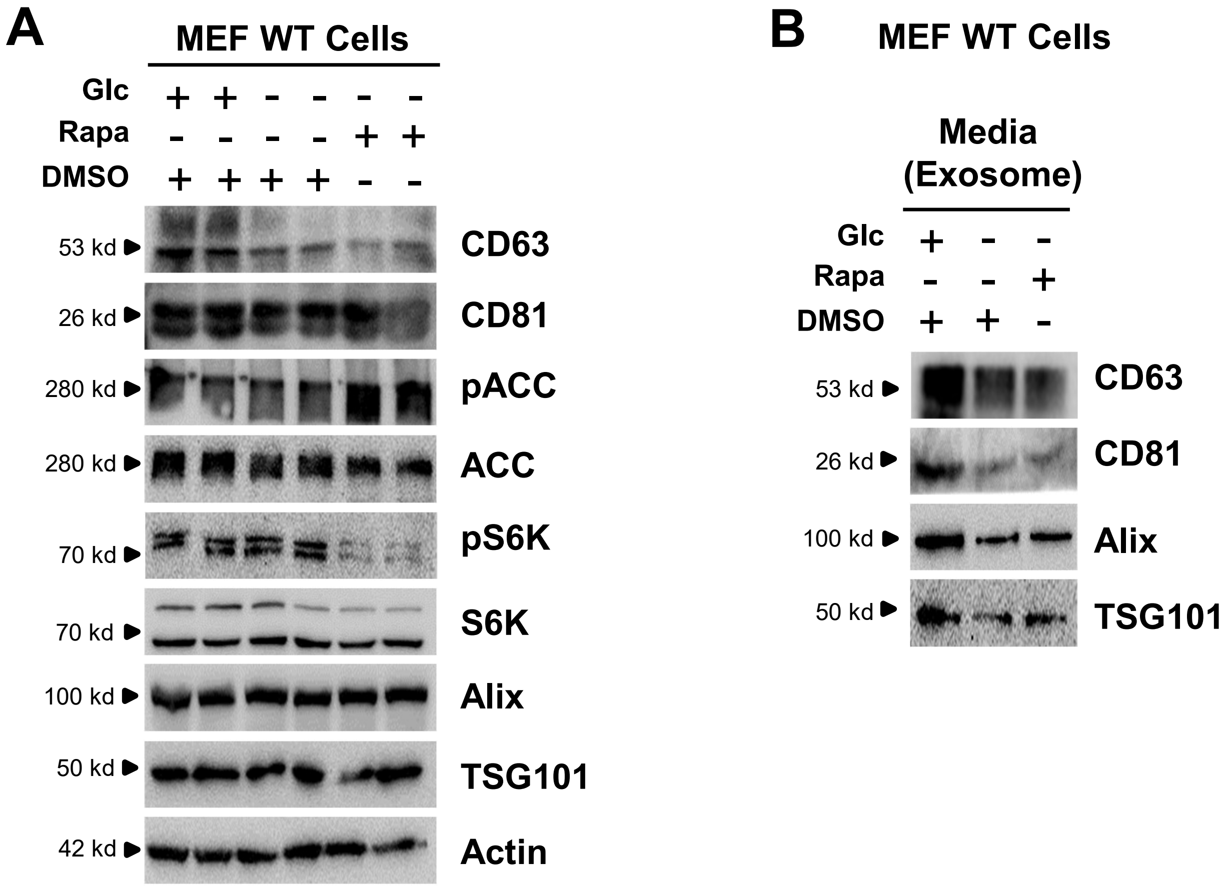

Supple. Figure S2. Effects of exosome release by mTORC1 inhibition in MEF-WT cells.

(A) MEF-WT cells were cultured with completed media. During serum starvation, cells were cultured in the absence or presence of glucose for 48 h. Rapamycin (100 nM) was treated in the absence of glucose for 24 h. The cells were harvested, lysed, and analyzed by immunoblotting with the indicated antibodies. Actin was used as a loading control. (B) MEF-WT cells were cultured with completed media. During serum starvation, cells were cultured in the absence or presence of glucose for 48 h. Rapamycin (100 nM) was treated in the absence of glucose for 24 h. After the collection of medium from cells, the media were subjected to ultracentrifugation to harvest the exosome fraction, as according to the materials and methods. The samples were analyzed by immunoblotting with antibodies for exosome marker proteins, such as CD63, CD81, Alix, and TSG101. Glucose (Glc), Rapamycin (Rapa), Dimethyl Sulfoxide (DMSO).

Supplemental Figure S2

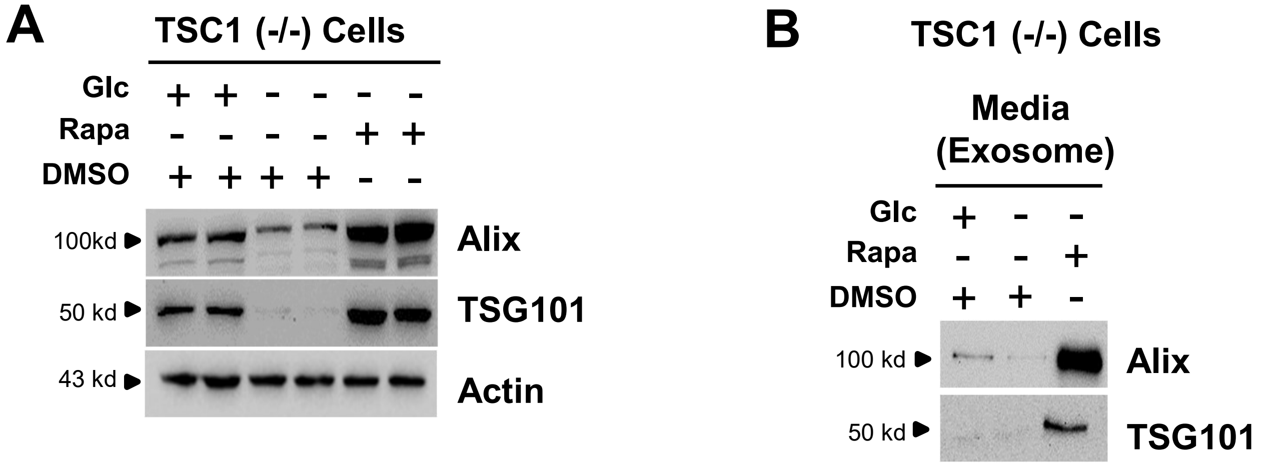

Supple. Figure S3. Effects of exosome release by mTORC1 inhibition in TSC1-null cells.
(A) TSC1 (-/-) MEF cells were cultured with completed media. During serum starvation, cells were cultured in the absence or presence of glucose for 48 h. Rapamycin (100 nM) was treated in the absence of glucose for 24 h. The cells were harvested, lysed, and analyzed by immunoblotting with the indicated antibodies such as Alix, and TSG101. Actin was used as a loading control. (B) TSC1 (-/-) MEF cells were cultured with completed media. During serum starvation, cells were cultured in the absence or presence of glucose for 48 h. Rapamycin (100 nM) was treated in the absence of glucose for 24 h. After the collection of medium from cells, the media were subjected to ultracentrifugation to harvest the exosome fraction, as according to the materials and methods. The samples were analyzed by immunoblotting with antibodies for exosome marker proteins, such as Alix, and TSG101. Glucose (Glc), Rapamycin (Rapa), Dimethyl Sulfoxide (DMSO).

Supplemental Figure S3

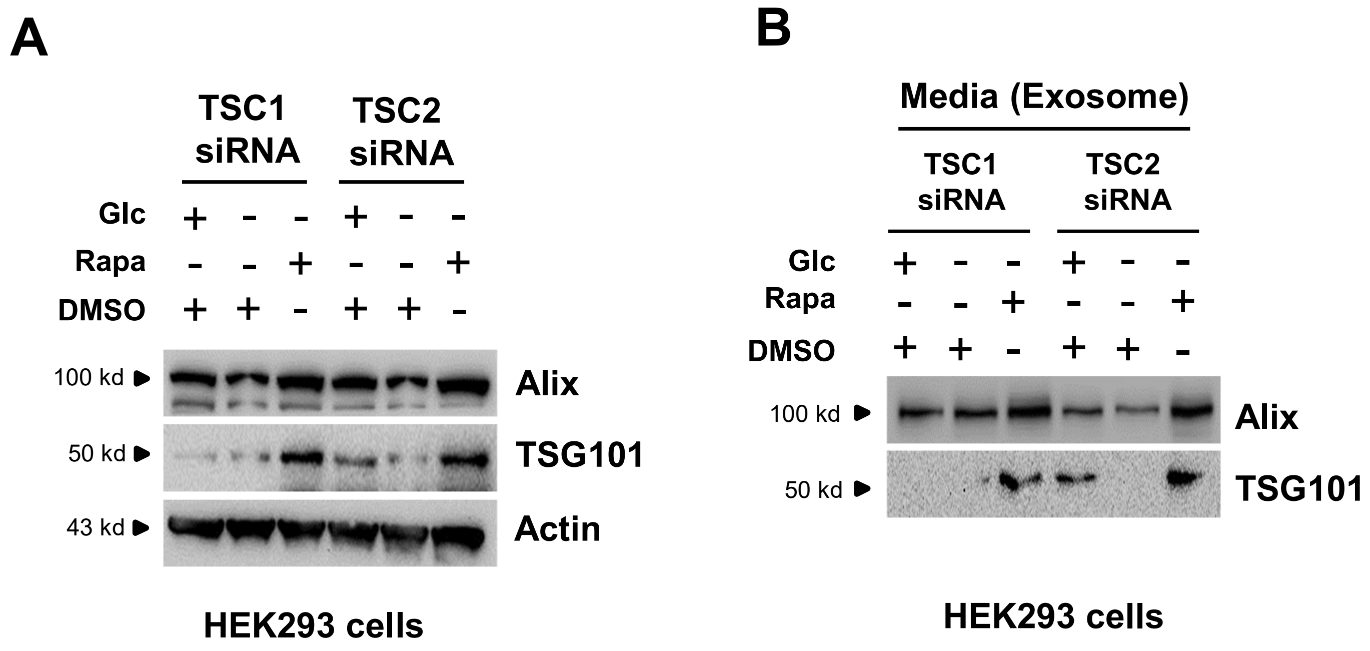

Supple. Figure S4. Effects of exosome release by mTORC1 inhibition in TSC1- or TSC2-silencing cells.
(A) HEK293 cells transfected with either TSC1 siRNA or TSC2 siRNA were cultured with completed media, and then incubated in the absence or presence of glucose for 48 h during serum starvation. The cells incubated in the absence of glucose were treated with 100 nM rapamycin for 24 h. The cells were harvested and lysed, and analyzed by immunoblotting with the indicated antibodies. Actin was used as a loading control. (B) HEK293 cells transfected with either TSC1 siRNA or TSC2 siRNA were cultured with completed media, and then incubated in the absence or presence of glucose for 48 h during serum starvation. After the collection of medium from cells, the media were subjected to ultracentrifugation to harvest the exosome fraction, according to the materials and methods. The samples were analyzed by immunoblotting with antibodies for exosome marker proteins, such as Alix, and TSG101. Glucose (Glc), Rapamycin (Rapa), Dimethyl Sulfoxide (DMSO).

Supplemental Figure S4

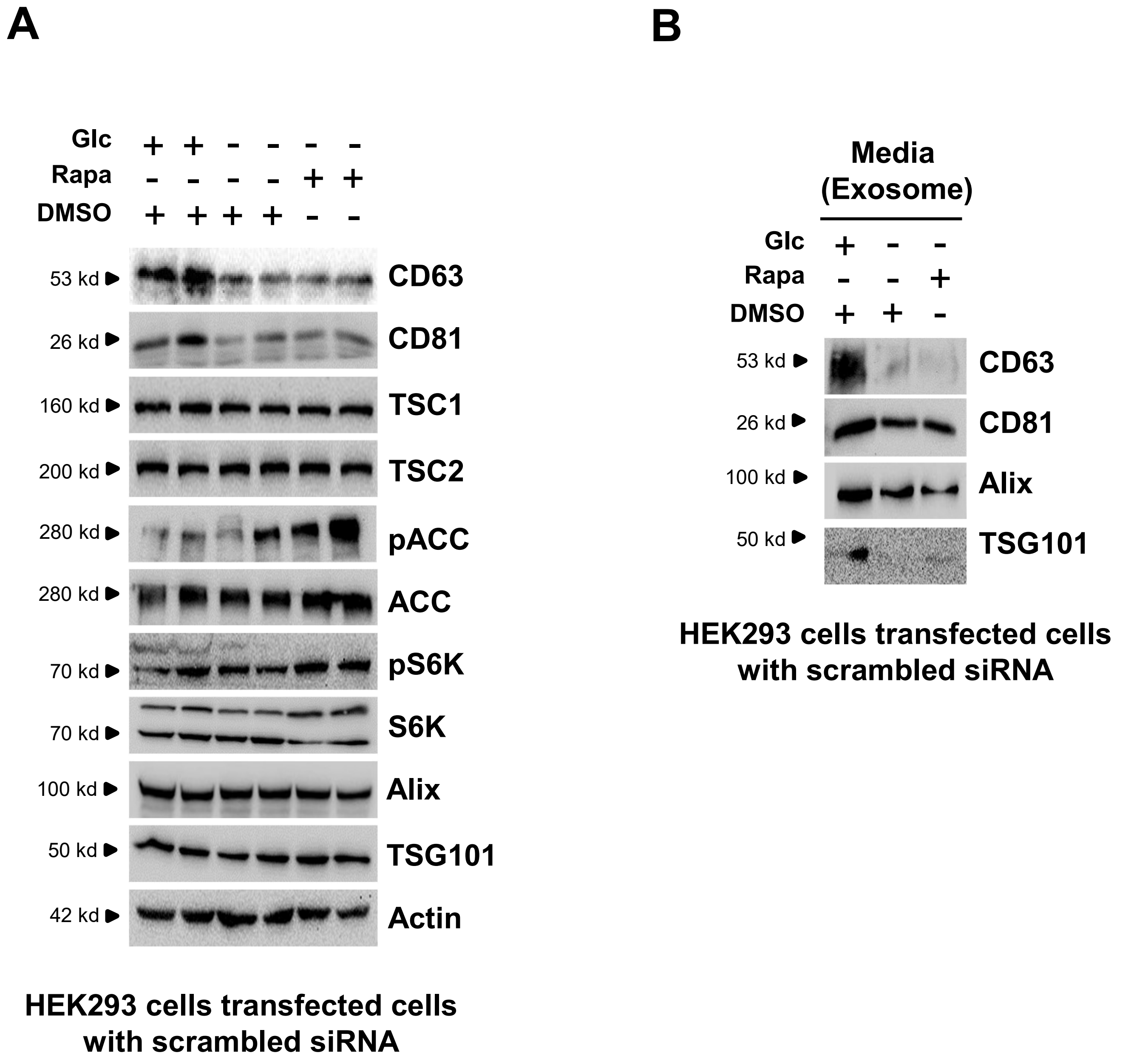

Supple. Figure S5. Effects of exosome release by mTORC1 inhibition in HEK293 cell transfected with scrambled siRNA.

(A) HEK293 cells transfected with scrambled siRNA were cultured with completed media, and then incubated in the absence or presence of glucose for 48 h during serum starvation. The cells incubated in the absence of glucose were treated with 100 nM rapamycin for 24 h. The cells were harvested and lysed, and analyzed by immunoblotting with the indicated antibodies. Actin was used as a loading control. (B) HEK293 cells transfected with scrambled siRNA were cultured with completed media, and then incubated in the absence or presence of glucose for 48 h during serum starvation. After the collection of medium from cells, the media were subjected to ultracentrifugation to harvest the exosome fraction, according to the materials and methods. The samples were analyzed by immunoblotting with antibodies for exosome marker proteins, such as CD63, CD81, Alix, and TSC101m. Glucose (Glc), Rapamycin (Rapa), Dimethyl Sulfoxide (DMSO).

Supplemental Figure S5

Figure 1A

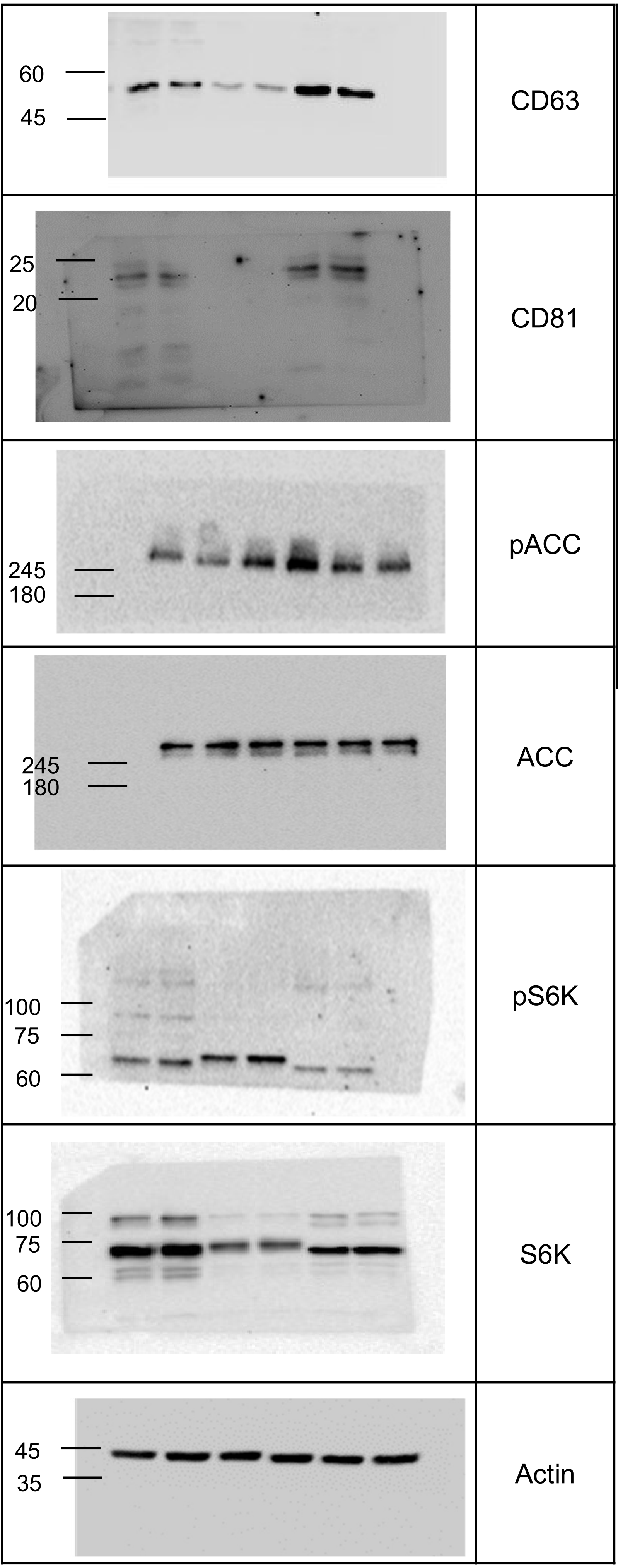

Figure 1C

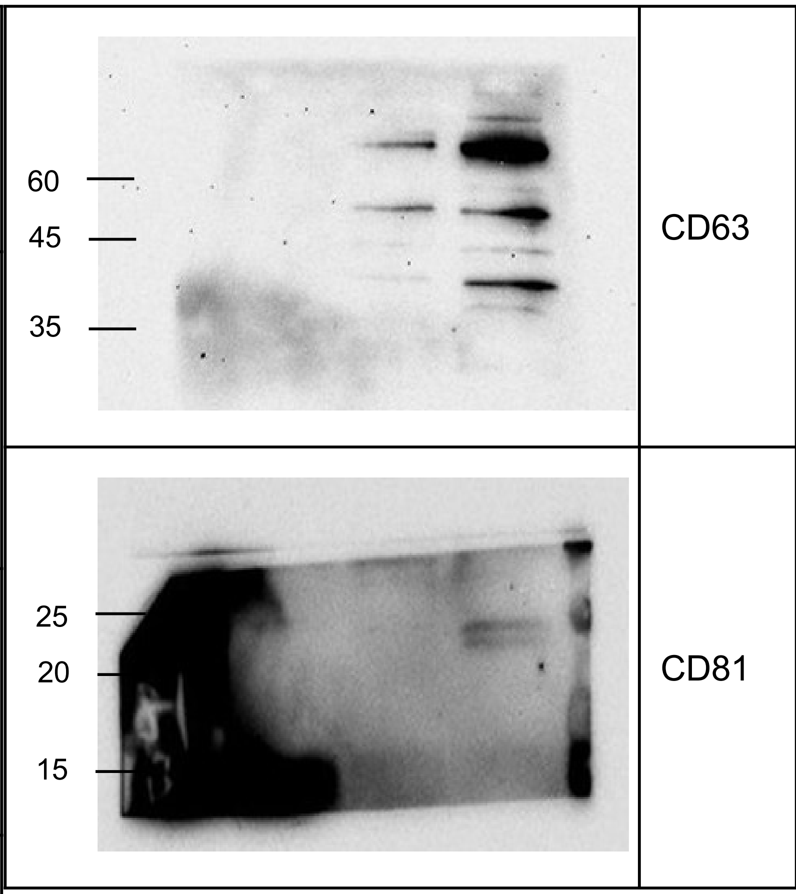

Supplemental Figure S6

Figure 2A

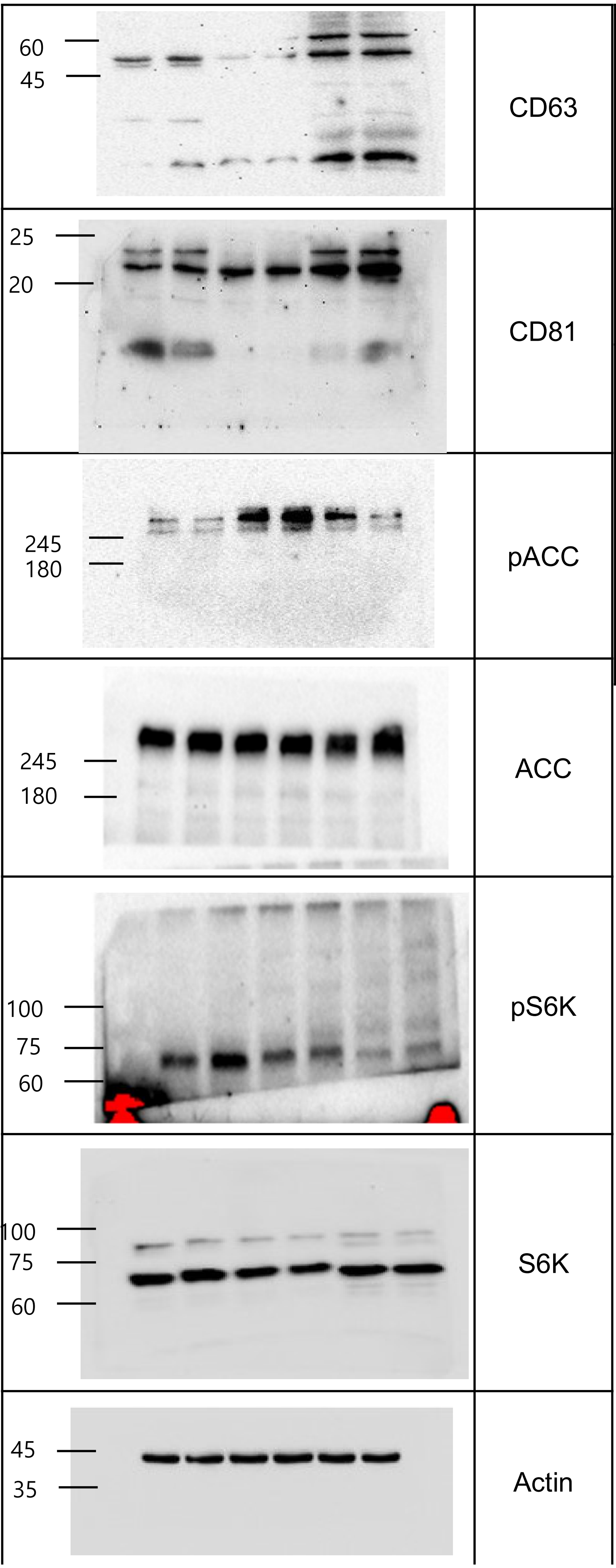

Figure 2C

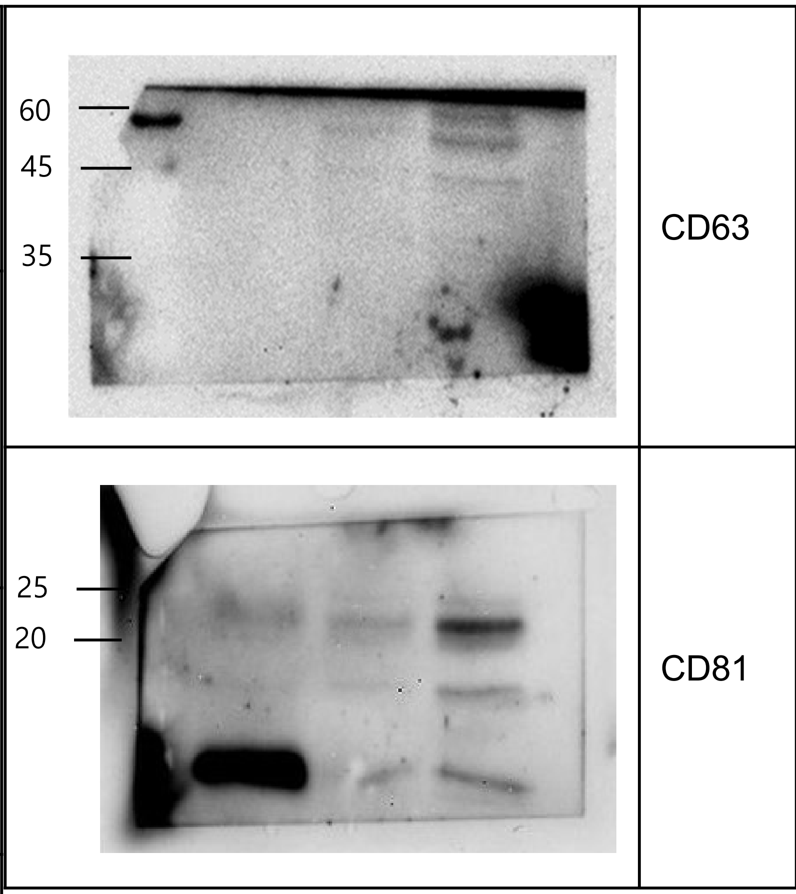

Supplemental Figure S6

Figure 3A

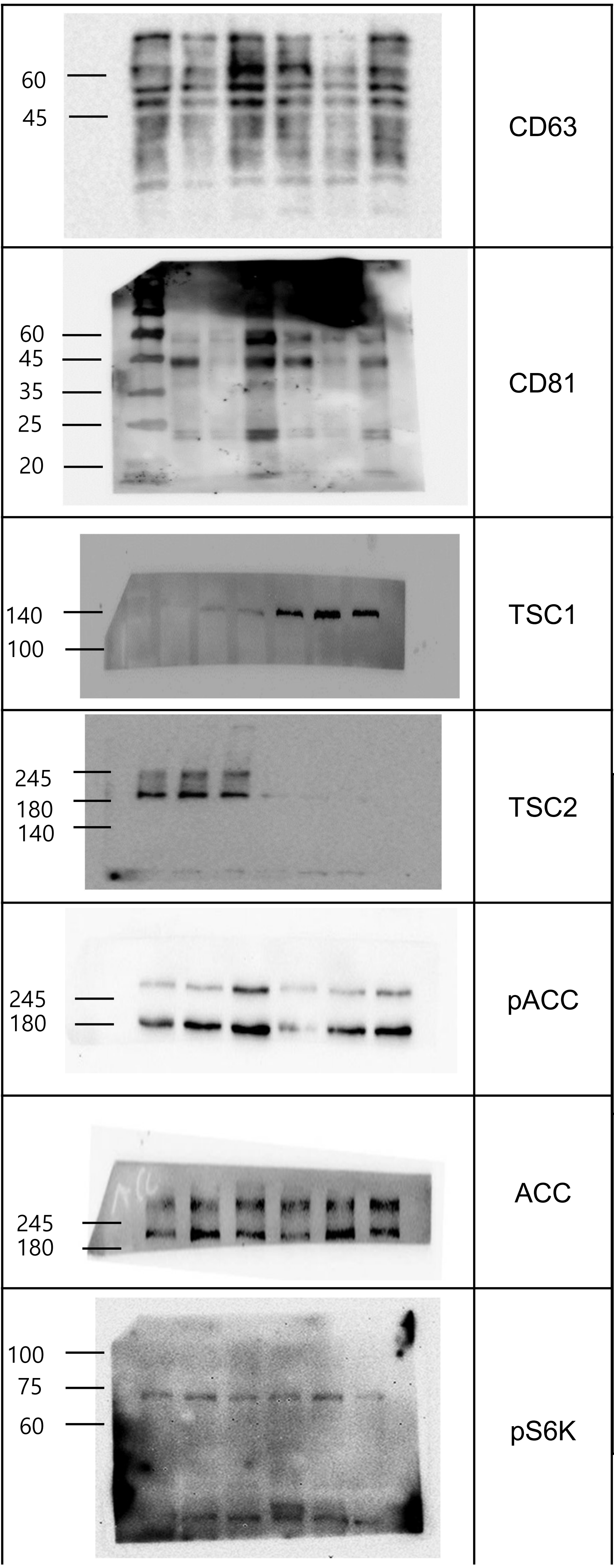

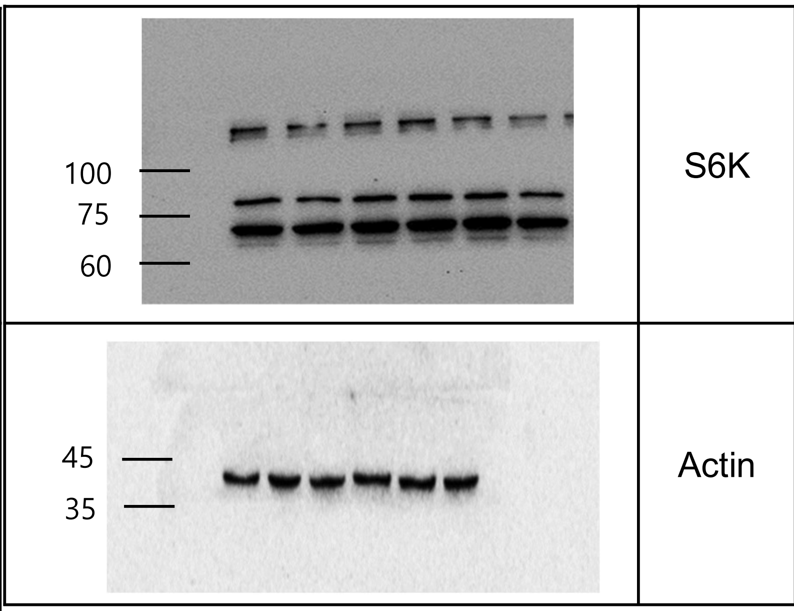

Figure 3C

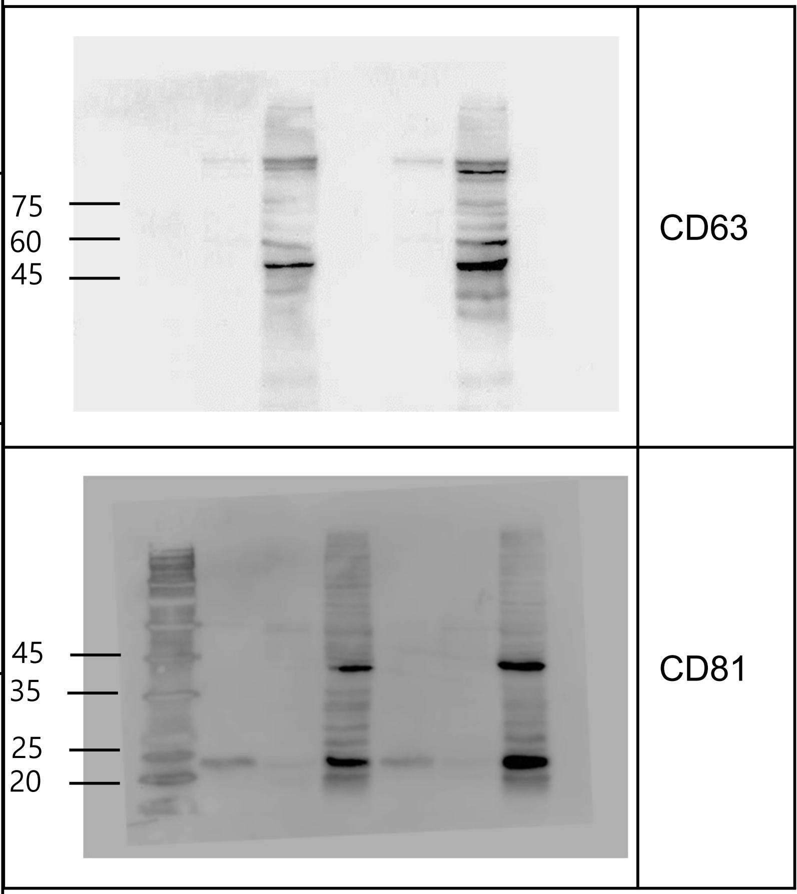

Supplemental Figure S6

Figure 4F

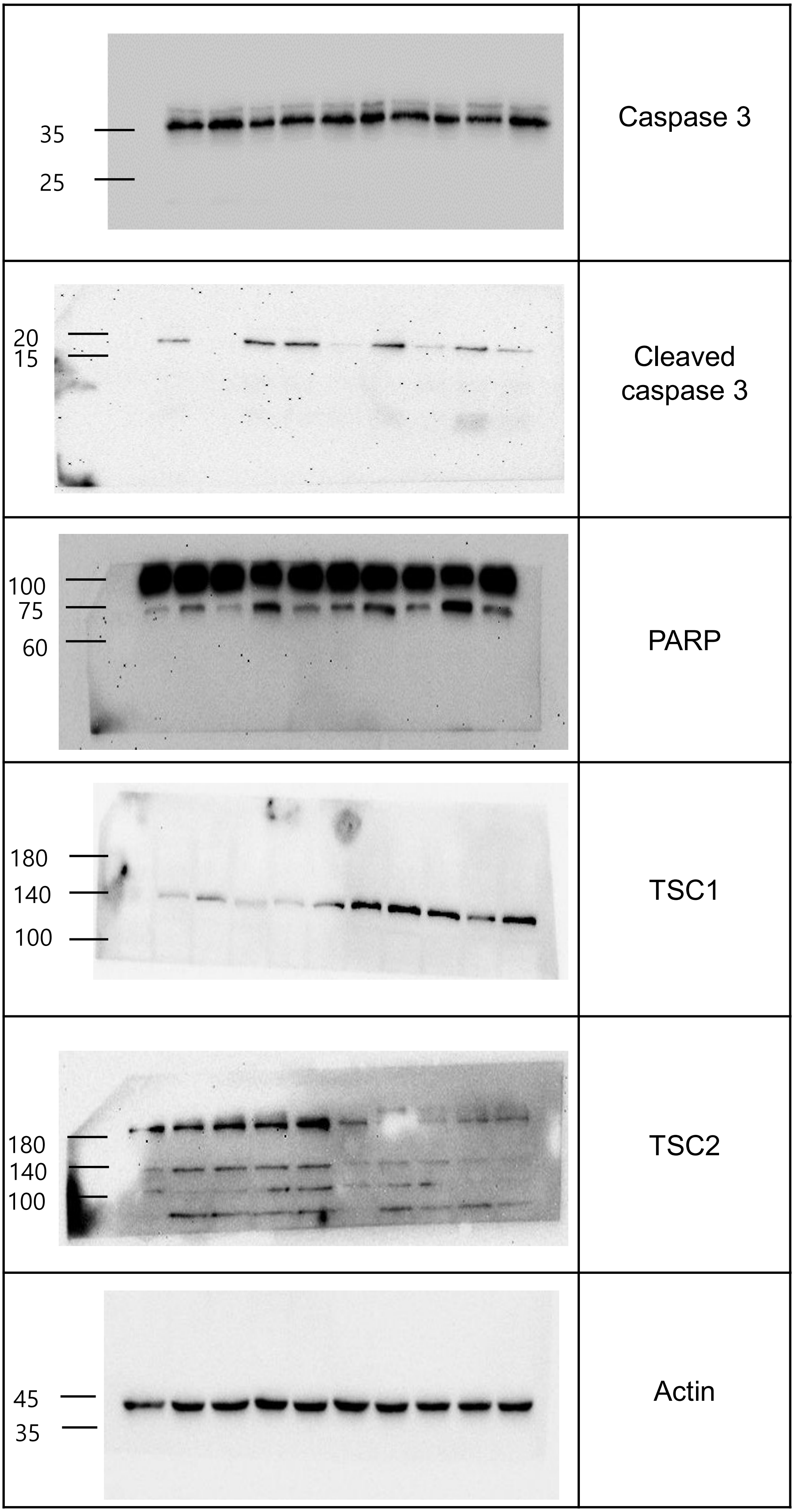

Supplemental Figure S6

Supple. Figure S1A

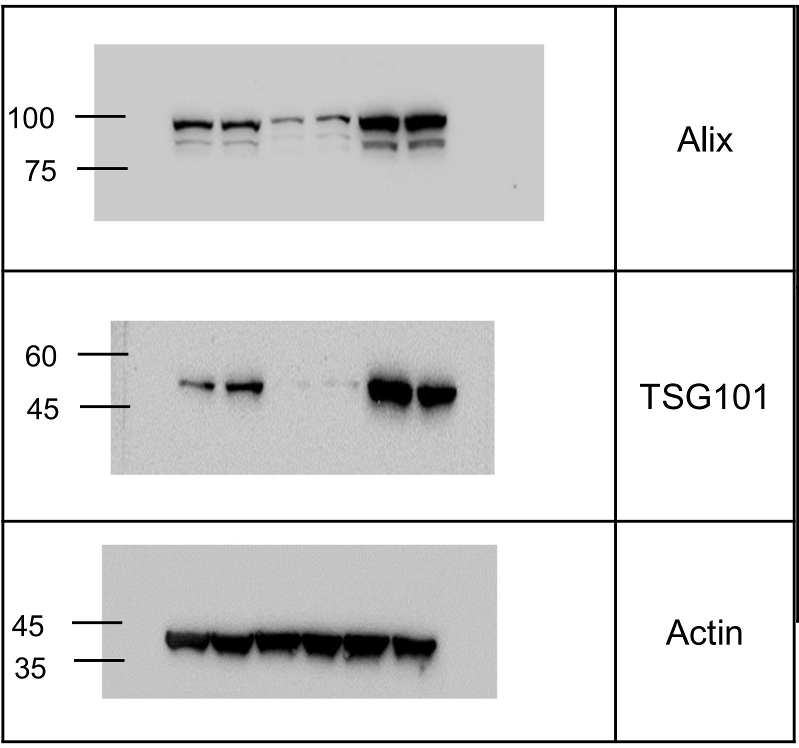

Supple. Figure S1B

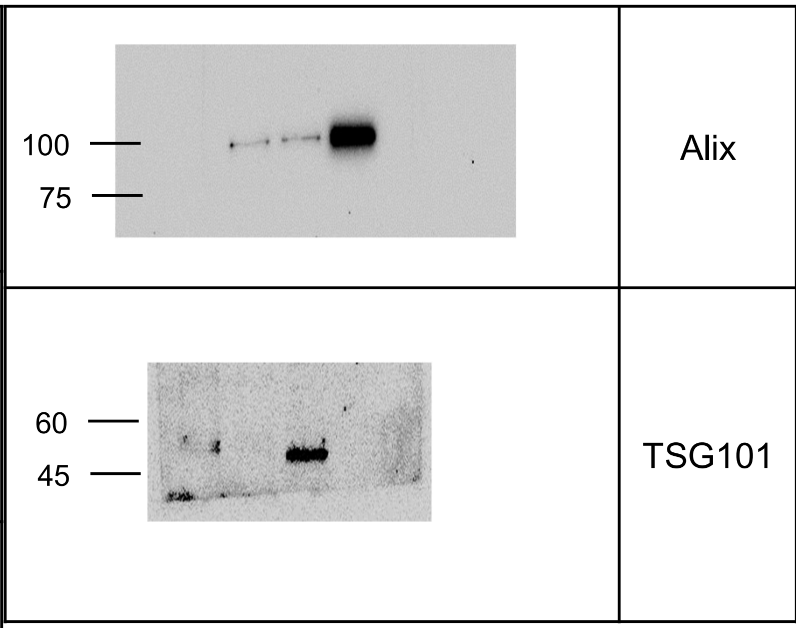

Supplemental Figure S6

Supple. Figure S2A

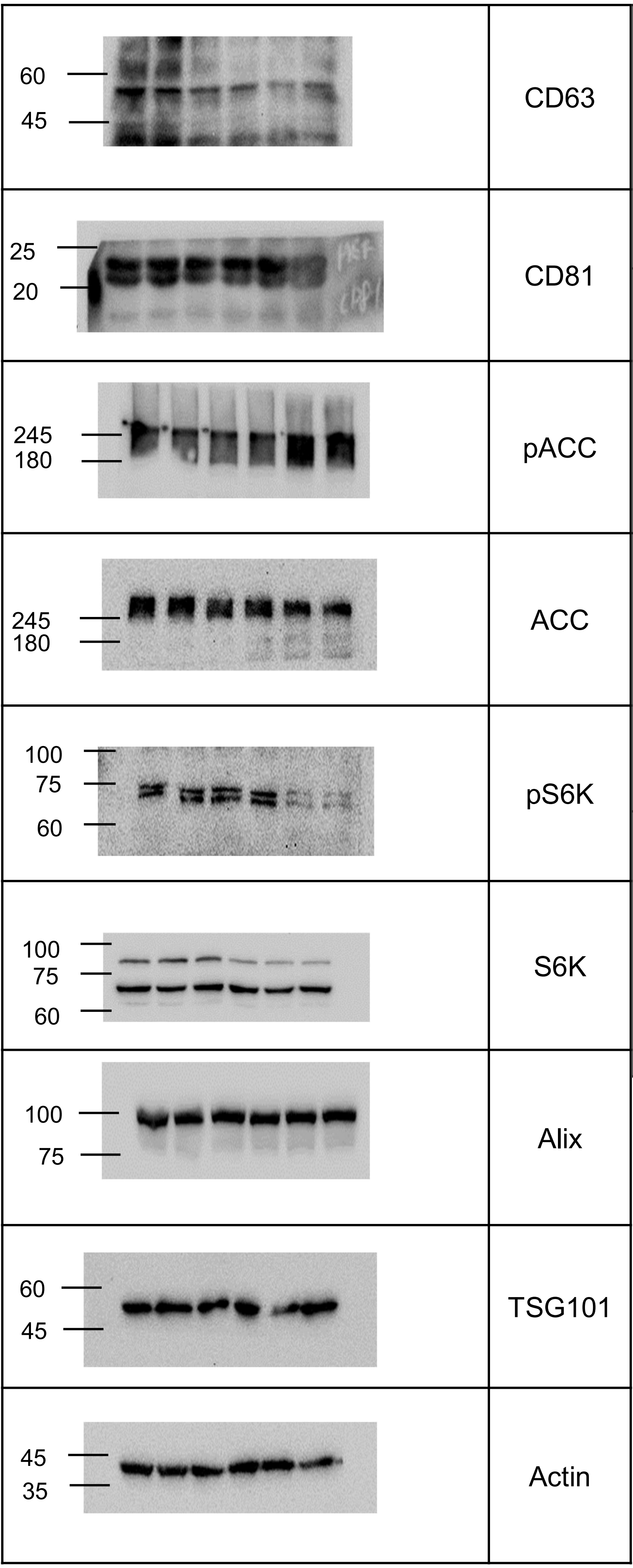

Supple. Figure S2B

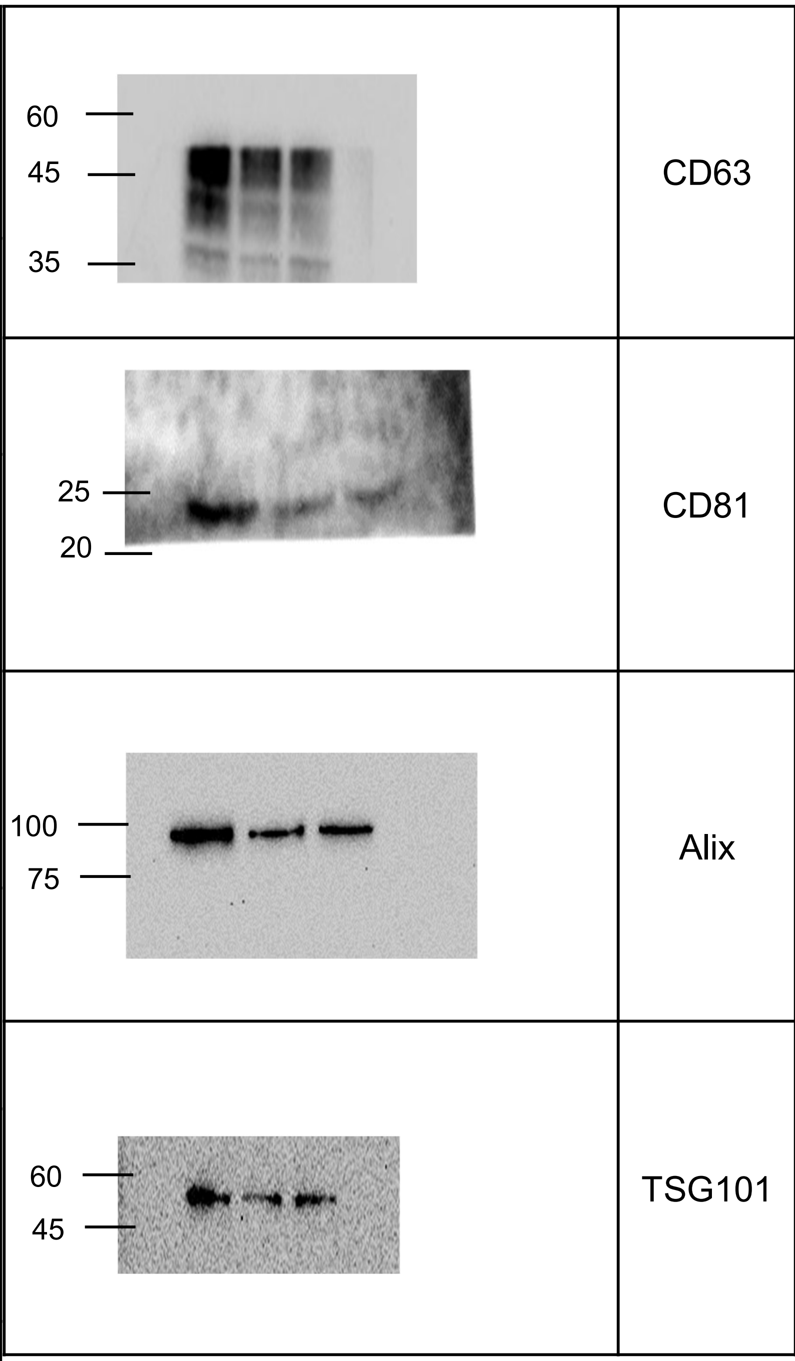

Supplemental Figure S6

Supple. Figure S3A

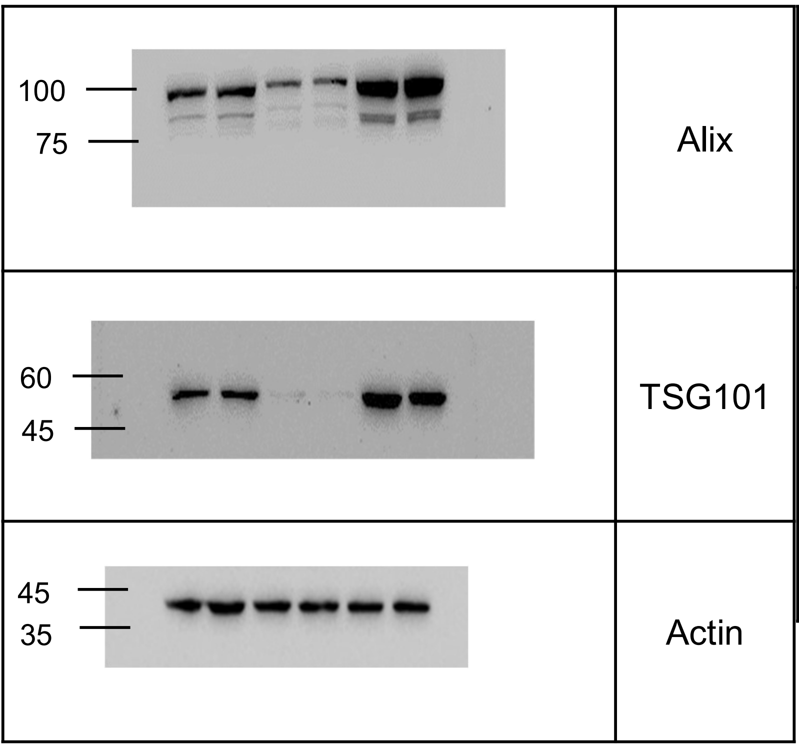

Supple. Figure S3B

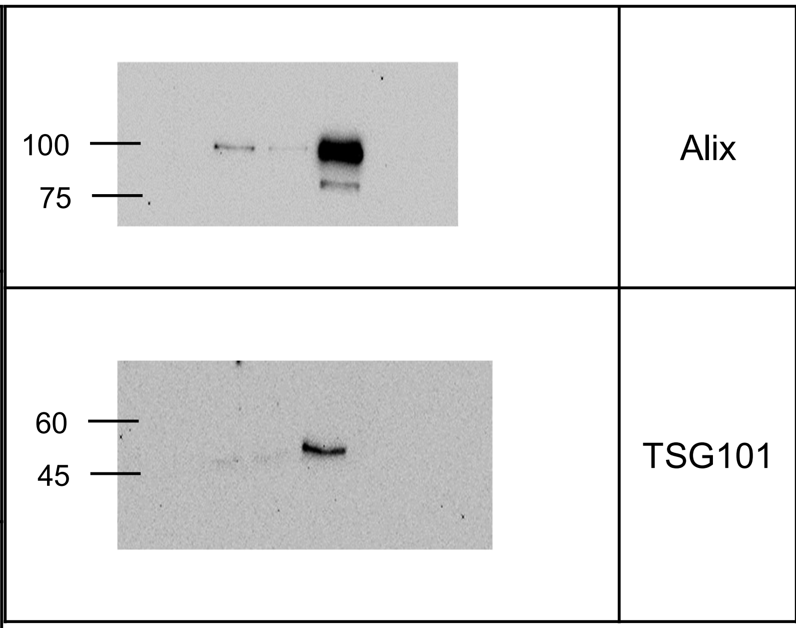

Supplemental Figure S6

Supple. Figure S4A

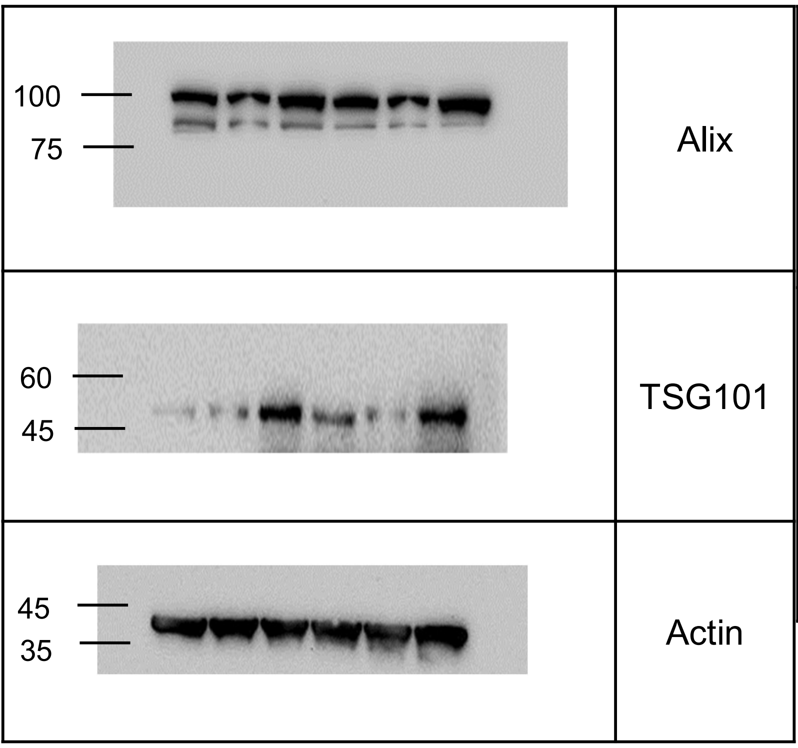

Supple. Figure S4B

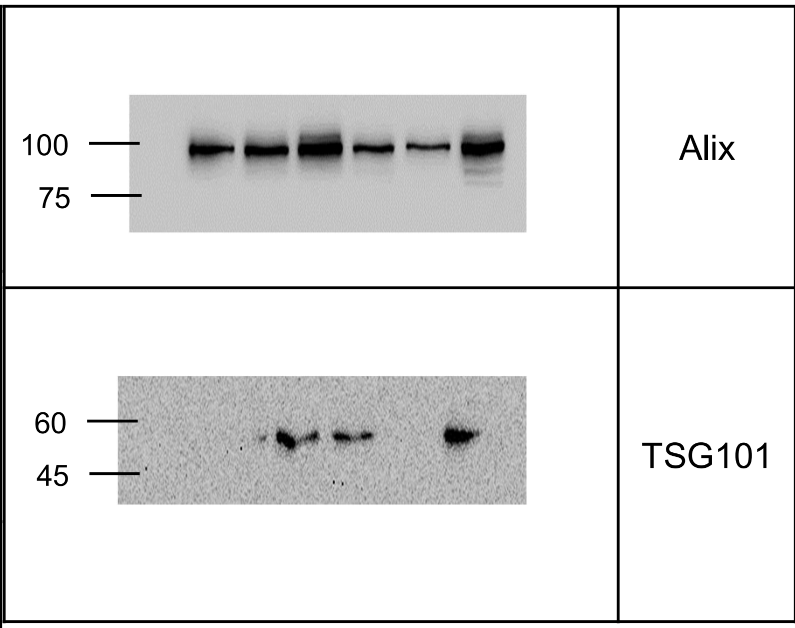

Supplemental Figure S6

Supple. Figure S5A

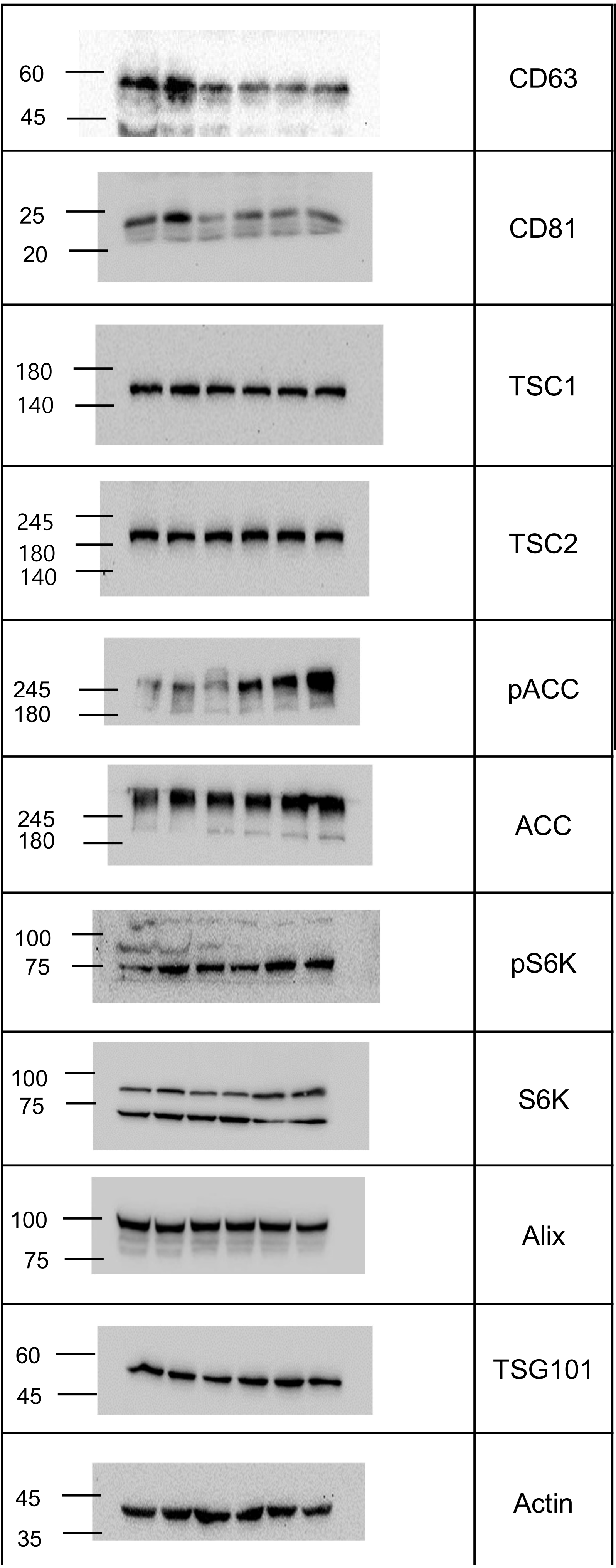

Supple. Figure S5B

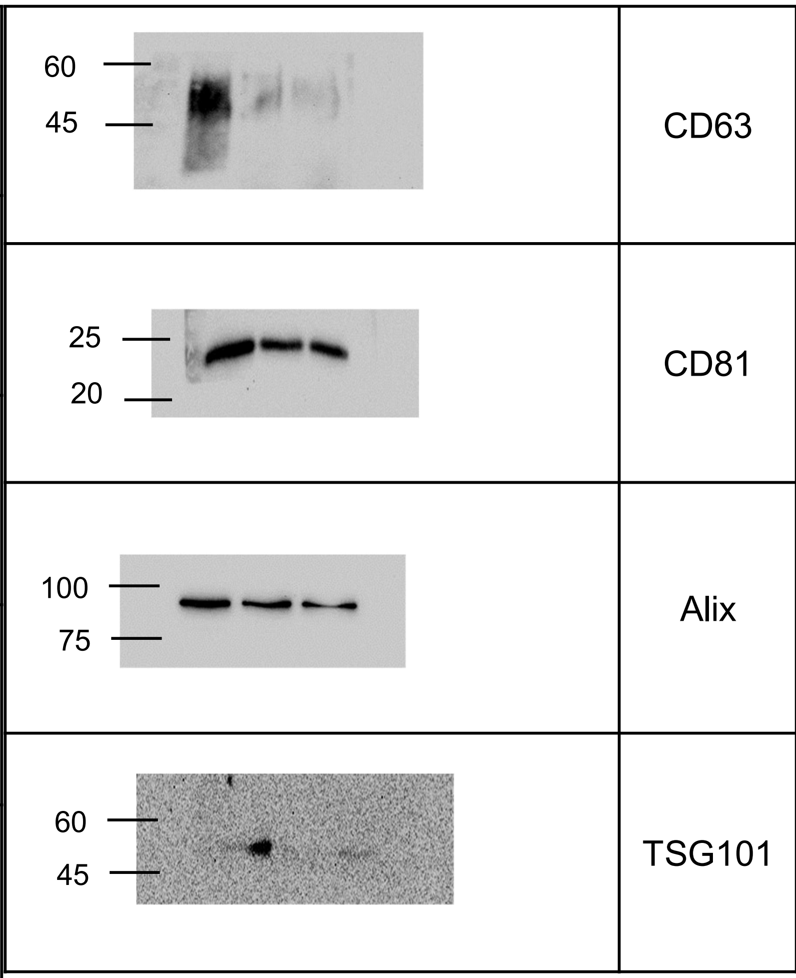

Supplemental Figure S6
